# Bioprinted 3D Primary Liver Tissues Allow Assessment of Organ-Level Response to Clinical Drug Induced Toxicity *In Vitro*

**DOI:** 10.1371/journal.pone.0158674

**Published:** 2016-07-07

**Authors:** Deborah G. Nguyen, Juergen Funk, Justin B. Robbins, Candace Crogan-Grundy, Sharon C. Presnell, Thomas Singer, Adrian B. Roth

**Affiliations:** 1 Organovo Holdings Inc., San Diego, CA, United States of America; 2 Roche Pharmaceutical Research and Early Development, Roche Innovation Center, Basel, Switzerland; Vrije Universiteit Brussel, BELGIUM

## Abstract

Modeling clinically relevant tissue responses using cell models poses a significant challenge for drug development, in particular for drug induced liver injury (DILI). This is mainly because existing liver models lack longevity and tissue-level complexity which limits their utility in predictive toxicology. In this study, we established and characterized novel bioprinted human liver tissue mimetics comprised of patient-derived hepatocytes and non-parenchymal cells in a defined architecture. Scaffold-free assembly of different cell types in an in vivo-relevant architecture allowed for histologic analysis that revealed distinct intercellular hepatocyte junctions, CD31+ endothelial networks, and desmin positive, smooth muscle actin negative quiescent stellates. Unlike what was seen in 2D hepatocyte cultures, the tissues maintained levels of ATP, Albumin as well as expression and drug-induced enzyme activity of Cytochrome P450s over 4 weeks in culture. To assess the ability of the 3D liver cultures to model tissue-level DILI, dose responses of Trovafloxacin, a drug whose hepatotoxic potential could not be assessed by standard pre-clinical models, were compared to the structurally related non-toxic drug Levofloxacin. Trovafloxacin induced significant, dose-dependent toxicity at clinically relevant doses (≤ 4uM). Interestingly, Trovafloxacin toxicity was observed without lipopolysaccharide stimulation and in the absence of resident macrophages in contrast to earlier reports. Together, these results demonstrate that 3D bioprinted liver tissues can both effectively model DILI and distinguish between highly related compounds with differential profile. Thus, the combination of patient-derived primary cells with bioprinting technology here for the first time demonstrates superior performance in terms of mimicking human drug response in a known target organ at the tissue level.

## Introduction

Drug-induced liver injury (DILI) is the leading cause of acute liver failure and post-market drug withdrawals. While the exact root cause of DILI often remains elusive and is usually multi-factorial, a common aspect is the interplay and cross-talk between the various cell types present within the liver [1–8]. For pre-clinical safety assessment of novel drug candidates, animal tests have typically been used and are still required as part of the data package provided to regulatory authorities. However, it is widely recognized that data generated in rodents translate only to a limited extent to humans and the relevance of such tests is being questioned—apart from the additional ethical aspects around animal use [9]. Significant efforts and resources therefore are expended during drug development to not only model the human liver accurately with respect to metabolism but also to predict and understand the mechanisms of DILI. Primary human hepatocytes are widely used as pre-clinical models of human liver to predict drug disposition endpoints and toxicity. However, the rapid loss of hepatocyte function ex vivo in traditional monolayer culture systems limits their application to short term acute effects [10, 11]. Over the last decade, researchers have demonstrated clear benefits from culture conditions that encourage three-dimensional growth, with hepatocytes displaying increased viability and functional capability when grown in sandwich cultures [12] or self-assembled spheroids [13]. Indeed, some functions of metabolism are transiently stabilized in these culture systems, including drug metabolism, transport, and CYP induction. In some cases, non-parenchymal cells (e.g. stellate, endothelial and immune cells) have also been added to the 2D or spheroid cultures. The resulting co-culture systems have demonstrated some enhanced functionality when compared to primary hepatocyte monolayer or sandwich cultures, including enhancement of hepatocyte viability and the ability to investigate the contribution of inflammatory mechanisms to toxicity [10, 11, 14, 15]. Beyond the impact of the multi-cellular context, spatial patterning has been shown to be critical both in normal liver physiologic function and in the pathophysiology of liver disease [16]. Hepatocytes that are located in the portal region naturally experience higher levels of nutrients and altered oxygen tension that result in spatially defined metabolic enzyme expression. As a consequence, toxic metabolites are produced and mediate tissue damage in distinct regions of the lobule, as has been demonstrated histologically in acute acetaminophen toxicity [17]. With this in mind, phenotypes that are a consequence of specific spatial patterns, such as cellular polarity and metabolite production, may suffer from issues of reproducibility in self-assembled *in vitro* systems. Recent advances in microfabrication techniques allow for controlled cell patterning, leading to defined heterotypic cell contacts and subsequent enhancement of hepatocyte functionality [18, 19]. Until recently, fabrication methods that enabled controlled spatial patterning of two or more cell types were limited to two-dimensional cultures or cultures that were a few cell layers thick [18]. While those techniques allow preservation of oxygen and nutrient diffusion, they also result in a higher proportion of contact with a solid surface versus cell-cell contact, which may accelerate loss of function in specialized cell types such as hepatocytes, and may result in aberrant responses to stimuli [20]. 3D bioprinting affords a means of fabricating tissue that is both spatially patterned and sufficiently three-dimensional (i.e., typically 200 microns or greater in the smallest dimension) such that it can be assessed histologically as well as biochemically [21, 22]. Histological examination remains a significant mode of diagnosis in the assessment of toxic outcomes *in vivo*, especially those in which physiologically-relevant doses are applied over extended periods of time and mechanism(s) of toxicity involve more than simple direct cytotoxicity to hepatocytes. Changes in relevant biomarkers in these cases are often not evident until well after the disease has been established, making it difficult to intervene early enough to halt progression [23, 24]. A fully human *in vitro* system comprising multiple liver cell types in a defined spatial architecture that can be used to gather both histopathological and biochemical data therefore has the potential to provide important insights about the human tissue response in the pre-clinical setting, before costly human trials are initiated.

To address these issues, bioprinted 3D human liver tissues composed of primary human parenchymal (hepatocyte) and non-parenchymal (endothelial and hepatic stellate) cell populations were evaluated for their potential use as durable, multi-cellular models of human liver tissue. Basic histologic, biochemical, and metabolic characterization of the 3D liver tissues was performed. In addition, to investigate the ability of the tissues to be used as a model of DILI, we tested their response to the known hepatotoxicant Trovafloxicin compared to its non-toxic relative Levofloxacin. Taken together, our results suggest that the 3D liver tissues could be a valuable addition to the pre-clinical toxicity pipeline.

## Materials and Methods

### Bioprinted 3D Liver tissues

3D Liver tissues comprised of cryopreserved primary human hepatocytes (Life Technologies, Carlsbad, CA, USA), hepatic stellates (ScienCell; Carlsbad, CA, USA), and HUVEC cells (Becton Dickinson; Tewksbury, MA, USA) were manufactured by Organovo (San Diego, CA, USA) using published patented protocols [25–28]. Briefly, stellate and HUVEC cells were propagated per the manufacturer’s instructions prior to tissue fabrication. Cryopreserved hepatocytes were thawed and prepared for use per the manufacturer’s instructions. Each commercial cell supplier provides assurances that the cells come from tissues collected in compliance with applicable laws and provided based on informed consent by the donors. Separate high density bio-inks comprising parenchymal cells (100% cellular paste, generated via compaction) or non-parenchymal cells (150e6 cells /mL formulated in NovoGel^®^ 2.0 Hydrogel; [29]) were prepared and loaded into separate heads of the NovoGen Bioprinter^®^ Instrument[21, 25–28] within a standard biosafety cabinet. A computer script was then executed to deposit the bioinks in a two-compartment planar geometry onto the membranes of standard 24-well 0.4 μm transwell culture inserts (Corning, Tewksbury, MA USA) via continuous deposition microextrusion (for in depth technology review, see [30]) with the non-parenchymal cells comprising the border regions of each compartment and the parenchymal cells filling each compartment. Following fabrication, the tissues were fed daily with 600 μL of 3D Liver Tissue Media, consisting of DMEM supplemented with Primary Hepatocyte Maintenance Supplements (Life Technologies, Carlsbad, CA, USA) and EGM-2 (Lonza, Basel, Switzerland),and incubated at 37°C under humidified atmospheric conditions supplemented with 5% CO2. Tissues were allowed to mature in culture for at least three days following fabrication prior to initiation of experimentation, and were substantially free of pre-formed scaffold at the time of use. For the one month timecourse, tissues were fed daily for a total of 28 days post-fabrication. For the CYP3A4 metabolism and compound toxicity studies, tissues were fed daily with or without treatments as described below.

### CYP3A4 Metabolism Assessment

**Fig 1:**
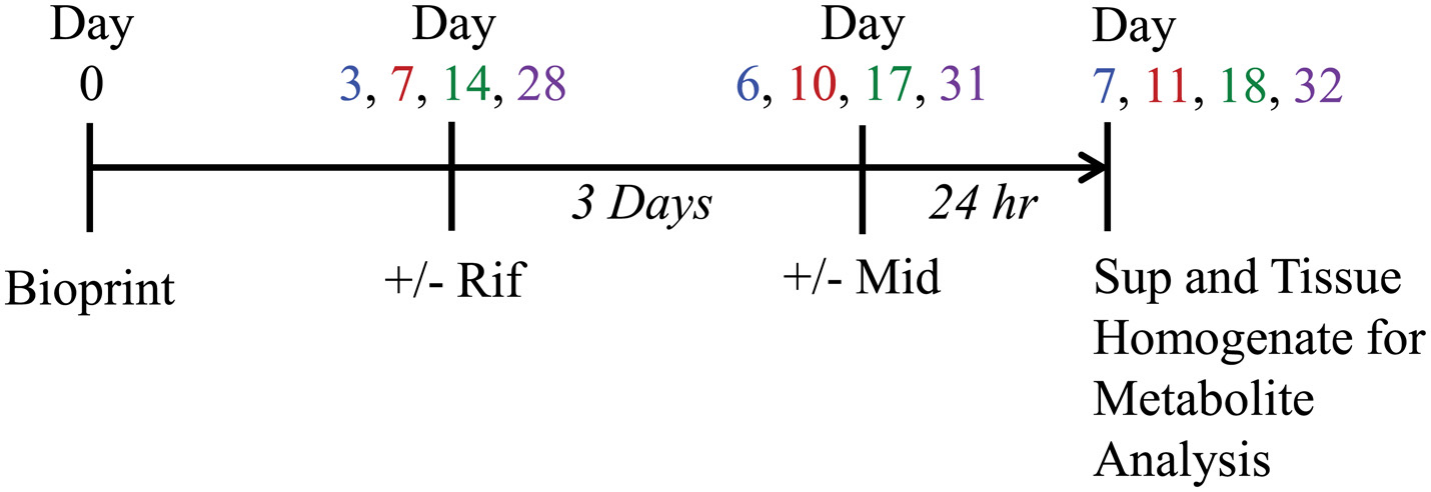
CYP3A4 Activity Experimental Timeline (Rif = Rifampicin, Mid = Midazolam).

To assess the expression and function of CYP3A4, bioprinted tissues were exposed to 10 μM Rifampicin (Rif; catalog# R3501-250MG, Sigma Chemical Company, St. Louis, MO, USA) or vehicle (0.1% DMSO) in 3D Liver Tissue Media, n = 6 per group, daily for 3 days as depicted on the experimental timeline below (Fig 1). Following 3 days of treatment with Rifampicin, spent media samples were collected and stored at -80°C as negative controls for metabolite formation, while 3 tissues treated with Rifampicin and 3 tissues treated with vehicle were homogenized in Trizol and stored at -80°C for later use in mRNA quantitation as described below. The remaining tissues (n = 3 with Rif, n = 3 with vehicle) were then exposed to Midazolam (Mid; catalog# M-908, Cerilliant Corporation). At 24 hours after treatment, the spent media samples were collected and frozen -80°C while the tissues were homogenized in PBS (without Mg and Ca), snap frozen on dry ice and stored -80°C. The frozen spent media and homogenized tissues in PBS were then analyzed for levels of 4-hydroxymidazolam using mass spec analysis done at SciAnalytical Strategies Inc. (La Jolla, CA, USA). Data shown is the average of 3 replicates plus or minus standard deviation.

### mRNA quantitation

Bulk RNA was extracted from 3D liver tissues at the time points indicated using Trizol (Life Technologies, Carlsbad, CA, USA) and Direct-zol RNA miniprep kit (Zymo, Irvine, CA, USA) per manufacturer’s instructions with the addition of tissue disruption using the Tissue Lyser (Qiagen, Valencia, CA, USA). RNA was quantified using the Nanodrop Lite (Thermo Scientific, Wilmington, Delaware USA). RT-PCR was carried out directly with RNA treated with DNase (Qiagen, Valencia, CA, USA). All RT-PCR amplification reactions were carried out using the StepOnePlus real-time PCR system (Life Technologies, Carlsbad, CA, USA) utilizing Fast 1-Step mix (Life Technologies, Carlsbad, CA, USA) and the following FAM-labeled Taqman gene expression assays (Life Technologies, Carlsbad, CA, USA): Hs00167927_m1 for CYP1A2, Hs02383631_s1 for CYP2C9, Hs00604506_m1 for CYP3A4, Hs03044634_m1 for CYP2B6, and Hs02576167_m1 for CYP2D6. VIC-labeled GAPDH endogenous control 4326317E was also amplified in the same well of each reaction for normalization. Fast RT-PCR reactions were conducted with the following conditions: reverse transcription at 50°C for 5 minutes; RT inactivation/denaturation at 95°C for 20 seconds; and 45 cycles of amplification at 95°C for 3 seconds followed by 60°C for 30 seconds. Data was expressed as delta Ct relative to GAPDH and converted to relative quantity (RQ) by the calculation RQ = [2^(- delta Ct)]*10,000. Data shown are the average of duplicate qPCR wells from 3 tissues per group plus or minus standard deviation and was graphed in Prism Software (Graphpad, La Jolla, CA, USA).

### Compound toxicity assays

**Fig 2:**
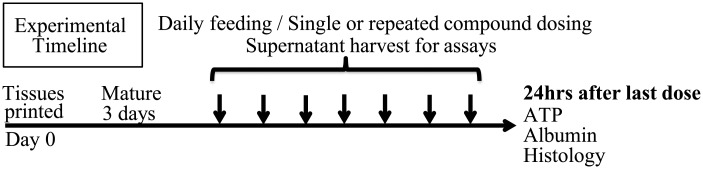
Experimental Timeline for Trovafloxacin toxicity studies.

For assessment of compound toxicity, Trovafloxacin mesylate (catalog# PZ0015-25mg; lot# 020M4708V) and Levofloxacin (catalog# 28266-10-F; lot# BCBF7004V) were purchased from Sigma Chemical Company (St. Louis, MO, USA). Stock compounds were serially diluted five-fold in dimethyl sulfoxide (DMSO) and then 5μl of stock compounds were diluted into 5ml of tissue 3D Liver Tissue Media^™^ to produce the working concentrations described at the same final concentration of DMSO at 0.1%. 5μl of DMSO was diluted into 5ml of 3D Liver Tissue Media^™^ to generate the vehicle control. Starting on day 3 of culture, bioprinted tissues were exposed to various concentrations of Trovafloxacin, Levofloxacin, vehicle, or media alone daily for 7 days as depicted on the experimental timeline below (Fig 2). Supernatants were collected daily and analyzed fresh for LDH activity. At 24 hours after the final dose, supernatants were collected and frozen at -80 for albumin analysis, while tissues were either lysed and analyzed for ATP content or fixed and embedded for histological characterization. Details of supernatant and tissue analyses are described below.

### Histological analysis using paraffin sections

At the timepoints described for each study, liver tissues were fixed in 2% paraformaldehyde solution (2% paraformaldehyde, 10mM calcium chloride, 50 mM sucrose in PBS) for 24 hours at room temperature. After 24 hours, the fixation solution was removed and replaced with 70% ethanol. Constructs with the attached transwell membrane were submerged in 45°C liquid Histogel (American MasterTech Scientific, Lodi, CA, USA) in a plastic biopsy mold and allowed to solidify at room temperature. Histogel blocks with embedded liver constructs were then processed for paraffin embedding using a tissue processor. Following infiltration with paraffin, liver constructs were embedded in paraffin molds and 5 μm cross-sections were prepared with a rotary microtome (Jung Biocut 2035; Leica Microsystems, Buffalo Grove, IL, USA). For hematoxylin and eosin (H&E) staining, slides were dewaxed in xylene and rehydrated through 100%, 95%, 70%, and 50% ethanol and rinsed in distilled water. Slides were immersed in Gill’s hematoxylin (Fisher Scientific, Pittsburgh, PA, USA). Following rinsing with distilled water, slides were briefly immersed in 0.2% v/v ammonium hydroxide. After rinsing with distilled water, slides were immersed in aqueous eosin solution (American MasterTech Scientific, Lodi, CA, USA). For Masson’s Trichrome staining, slides were deparaffinized and stained according to the manufacturer’s protocol (American MasterTech Scientific). Slides were then dehydrated through an ethanol gradient, cleared in xylene, and mounted with resinous mounting media (CytoSeal; Fisher Scientific, Pittsburgh, PA, USA).

For immunohistochemical analyses, slides were de-waxed in xylene and rehydrated through graded ethanols before finally washing in distilled water. Rehydrated sections were subjected to heat-mediated antigen retrieval in 10 mM sodium citrate pH 6.0 using a standard microwave oven to heat the solution and slides to a 95°C followed by slow cooling for 30 minutes. Slides were then blocked with 10% goat serum in Tris-buffered saline (TBS) for 1 hour, followed by incubation with primary antibodies overnight at 4°C. The following primary antibodies were utilized: rabbit anti-E-cadherin (1:50 dilution; Abcam, Cambridge, MA, USA); mouse anti-albumin (1:200 dilution; Sigma, St. Louis, MO, USA); rabbit anti-desmin (1:200 dilution; Abcam, Cambridge, MA, USA); mouse anti-CD31 (1:25 dilution; Abcam, Cambridge, MA, USA); rabbit anti-CD31 (1:250 dilution; Abcam, Cambridge, MA, USA); mouse-anti-smooth muscle actin (α-SMA, 1:200; Abcam, Cambridge, MA, USA); mouse anti—proliferating cell nuclear antigen (1:10,000 dilution; Abcam, Cambridge, MA, USA); rabbit anti-proliferating cell nuclear antigen (1:2000 dilution; Cell Signaling Technologies, Danvers, MA, USA). Sections were then washed three times in TBS with 0.1% Tween 20 and incubated with AlexaFluor 488 or AlexaFluor 568-conjugated secondary antibodies (Life Technologies, Carlsbad, CA, USA) diluted 1:200 in TBS. For α-SMA detection, fluorescence was applied using Tyramide signal amplification (TSA) Kit with HRP-streptavidin and Alexa Fluor 594 substrate (Thermo-Fisher Scientific, Pittsburgh, PA), for 1 minute each. Sections were then washed three times in TBS-0.1% Tween 20, rinsed with distilled water, and mounted with DAPI-containing mounting media (Vector Labs, Burlingame, CA, USA). Immunohistochemistry for vimentin was performed on the Ventana Discovery XT^®^ immunostainer (Ventana, Tucson, AZ, USA) with an anti-vimentin mouse monoclonal antibody (1:10 dilution; Ventana, Tucson, AZ, USA, 790–2917) as primary antibody and biotin-SP-conjugated donkey anti-mouse IgG (1:100 dilution; Jackson ImmunoResearch, West Grove, PA, USA, 715-065-151) in a standard protocol using the Ventana DAB Map^®^ kit (Ventana, Tucson, AZ, USA, 05266360001). Slides were counterstained with hematoxylin.

### Histological analysis using frozen sections

At the timepoints described for each study, liver constructs were rinsed once with DPBS, immersed in Tissue-Tek OCT compound (Sakura Finetek Europe B.V., The Netherlands), and flash frozen. Frozen blocks containing the liver construct were then sectioned at 5 μm on a cryostat (Leica Cryocut 1800, Leica Microsystems, Buffalo Grove, IL, USA). Sectioned slides were snap fixed in -20°C liquid acetone and allowed to air dry for 20 minutes at room temperature. For Oil Red O staining, slides were rehydrated in distilled water and immersed in 60% isopropanol for 2 min. Slides were stained with 0.3% w/v Oil Red O (Sigma, St. Louis, MO, USA) in 60% isopropanol for 15 minutes at room temperature, followed by rinsing with 60% isopropanol for 1 minute. Slides were immersed briefly in Gill’s hematoxylin to counterstain. Slides were rinsed with distilled water and mounted with aqueous media (American MasterTech Scientific, Lodi, CA, USA).

### Image acquisition

H&E, PAS, and Oil Red O-stained slides were imaged using a Zeiss Axioskop microscope (Zeiss, Jena, Germany). Images were acquired with an Insight 2 camera and Spot 5.0 software (Diagnostic Instruments, Inc., Sterling Heights, MI, USA). Fluorescently labeled slides were imaged with a Zeiss AxioImager microscope and images were acquired with a Zeiss ICM-1 camera and Zeiss Zen Pro software.

### Immunoassays

Supernatants frozen at the indicated study timepoints were thawed on ice and analyzed by ELISA for albumin (Bethyl Laboratories, Montgomery, TX, USA), per the manufacturer’s instructions on a microplate reader (BMG Labtech, Germany) with minor modifications. Briefly, a half-area 96-well plate was employed, and the volumes of the kit reagents were reduced by 50%. For the one month tissue timecourse, data shown is the average of 5 tissue replicates plus or minus standard deviation. In the case of the toxicity studies with Trovafloxacin and Levofloxacin, data shown is the average of 10 tissue replicates across two independent experiments plus or minus standard deviation.

### LDH assays

Supernatants for LDH were collected at the indicated timepoints for each study and analyzed fresh for LDH activity colorimetrically with a commercially available reagent (Abcam, Cambridge, MA, USA) per the manufacturer’s instructions with minor modifications. Briefly, a half-area 96-well plate was employed, allowing the volumes of the kit reagents to be reduced by 50%, and samples were diluted to obtain readings in the linear range of the standard curve. Data shown is the average of 10 tissue replicates across two independent experiments plus or minus standard deviation.

### Tissue viability assays

At the timepoints described for each study, tissues were lysed and analyzed for ATP content using Cell Titer Glo (Promega, Madison, WI, USA). Tissues were shaken in the presence of Cell Titer Glo reagent for 2 min then the mixture was transferred to 1.5mL Eppendorf tubes and triturated to further break up the tissues. Samples were allowed to equilibrate for 10 minutes at room temperature. Samples were centrifuged at 1000 g for 2 minutes and aliquots of supernatant were transferred to a white opaque 96 well plate for luminescence measurement on a microplate reader (BMG Labtech, Germany). For the one month tissue timecourse, data shown is the average of 3 replicates plus or minus standard deviation. In the case of the toxicity studies with Trovafloxacin and Levofloxacin, data shown is the average of 6 tissue replicates across two independent experiments plus or minus standard deviation.

### 2D Hepatocyte Cultures

Cryopreserved human hepatocytes were thawed according to manufacturer protocol and seeded on collagen I coated 24 well plates (Becton Dickenson; Tewksbury, MA, USA) at a density of 4.23e5 cells per well in primary hepatocyte plating media (Life Technologies, Carlsbad, CA, USA). Hepatocytes were incubated at 37°C with 5% CO2 to enable attachment for 4 hours. Following the attachment incubation period the plating media was aspirated from the wells and cells were fed with hepatocyte maintenance media (Life Technologies, Carlsbad, CA, USA). Cells were incubated in maintenance media for the time period indicated, with daily media exchange. To assess Trovafloxacin toxicity in 2D hepatocyte cultures, media containing vehicle control or Trovafloxacin was prepared as described above for 3D tissue compound toxicity studies and dosed daily for 7 days prior to assessment of cell viability and albumin production. Viability assays (Cell Titer Glo; Promega, Madison, WI, USA) and albumin ELISA (Bethyl Laboratories, Montgomery, TX, USA) were performed as described above.

## Results and Discussion

### Histological characterization of 3D liver tissues

**Fig 3:**
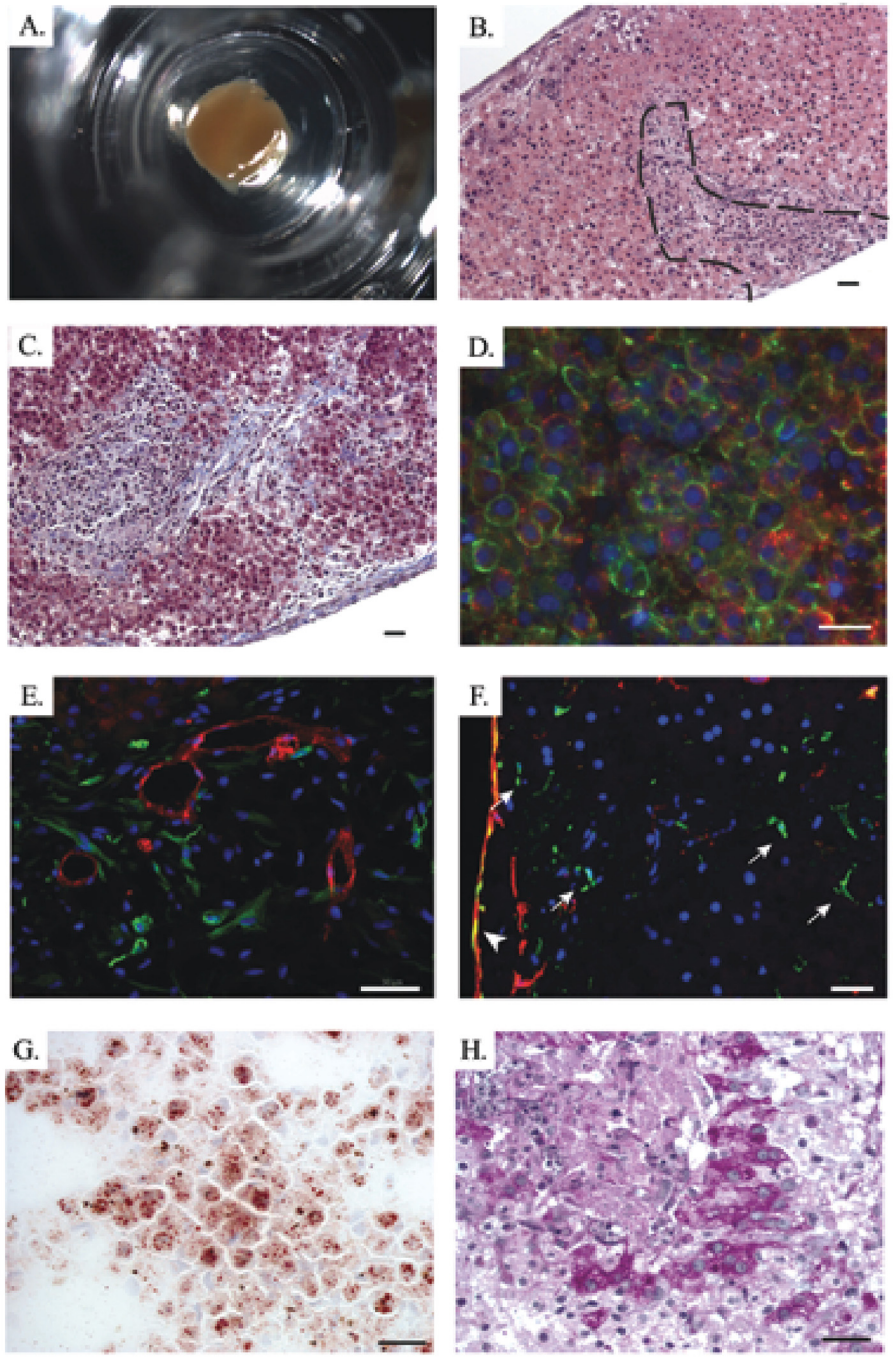
Histological characterization of 3D liver tissues. A) A macroscopic image of a 3D liver tissue housed in a 24 well transwell. B) H&E staining of a tissue cross-section; compartmentalization between the parenchymal and non-parenchymal fractions can be readily visualized (dashed line). C) ECM deposition assessed by Masson’s trichrome staining. D) IHC staining of the parenchymal compartment for E-cadherin (Green) and Albumin (red). E) IHC staining for CD31 (red) and desmin (green) to assess organization of the endothelial cells and the presence of quiescent hepatic stellates in the non-parenchymal compartment. F) IHC staining for desmin (green) and α-SMA (red) to assess stellate cell activation. White arrows indicate quiescent stellates in the tissue interior that stain positive for desmin and negative for α-SMA. Cells at the tissue periphery stain positive for α-SMA (white arrowhead), suggesting they have a more activated phenotype. G) Oil-red O staining of 3D liver tissue cryosections to measure lipid storage. H) PAS staining to identify glycogen granules. DAPI was utilized to stain the nuclei of the cells in all of the IHC staining samples (Blue). Scale bars in the lower right hand corner of images are 25μm (B-D, G-H) or 50μm (E, F).

**Fig 4:**
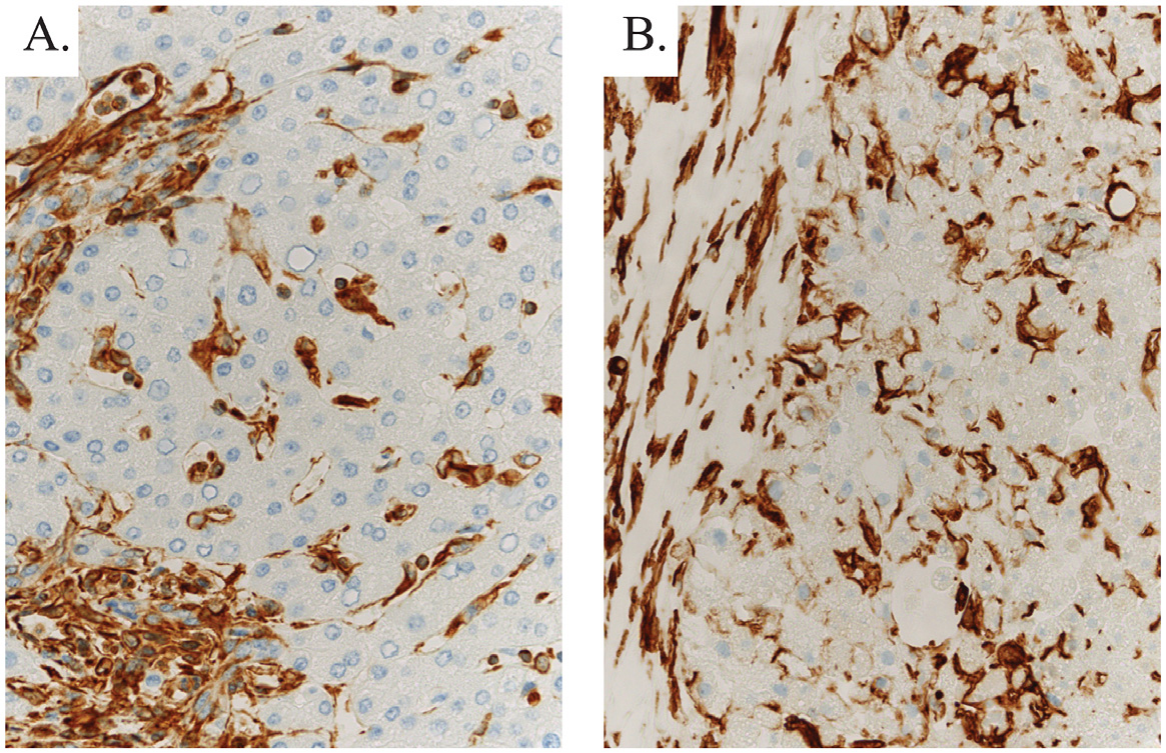
Immunohistochemical assessment of vimentin in bioprinted 3D liver tissues and native human liver. A) IHC staining for vimentin (brown) to assess the non-parenchymal (mesenchymal) compartment in human liver tissue and B) 3D liver tissues. Parenchymal cells are intermingled with vimentin-positive mesenchymal cells.

3D liver tissues composed of human hepatic stellate cells (HSC), human umbilical vein endothelial cells (HUVEC), and cryopreserved primary human hepatocytes were fabricated directly into 24-well Transwell^®^ plates (Fig 3A). Histologic analyses conducted throughout the maturation and maintenance period show that the bioprinted tissues retained the compartmentalization of parenchymal and non-parenchymal components established at the time of fabrication, and that the tissues condense and remodel over time, yielding stable 3D structures with a minimal thickness of 250 μm in the Z axis, dense tissue-like cellularity, and no evidence of necrosis (Fig 3B). Masson’s trichrome staining of the 3D bioprinted liver tissues revealed defined areas of collagen deposition in the non-parenchymal regions of the tissues, consistent with published evidence that functional endothelial cells produce and secrete extracellular matrix (ECM) during vasculogenesis [31] (Fig 3C). This ECM allows for the formation of a cohesive tissue unit without the use of exogenous scaffolding materials. Immunofluorescent analyses of the tissues showed robust surface expression of the intercellular junctional protein E-cadherin as well as cytoplasm-localized human albumin in the hepatocytes of the parenchymal compartment (Fig 3D). Over time in culture, the endothelial cells form extensive networks, with evidence of lumens by day 21 (Fig 3E). While human and rodent HSCs maintained in standard monolayer culture on plastic undergo activation to a myofibroblast phenotype with concomitant upregulation of smooth muscle actin (α-SMA), the HSC resident in the interior of the 3D liver tissues express desmin and not α-SMA (Fig 3E and 3F), suggesting that they are capable of establishing and maintaining a quiescent state within the 3D multicellular environment [32, 33]. A subpopulation of activated stellate cells, identified by their expression of α-SMA, can be identified at the tissue/media interface (Fig 3F). Lipid storage and glycogen storage, two functions associated with hepatocytes in vivo, were also demonstrated in the bioprinted liver tissues after maturation (Fig 3G and 3H). Comparison of vimentin staining between bioprinted liver tissues and native human liver biopsy shows similar patterns of expression (Fig 4).

### Biochemical characterization of 3D liver tissues

**Fig 5:**
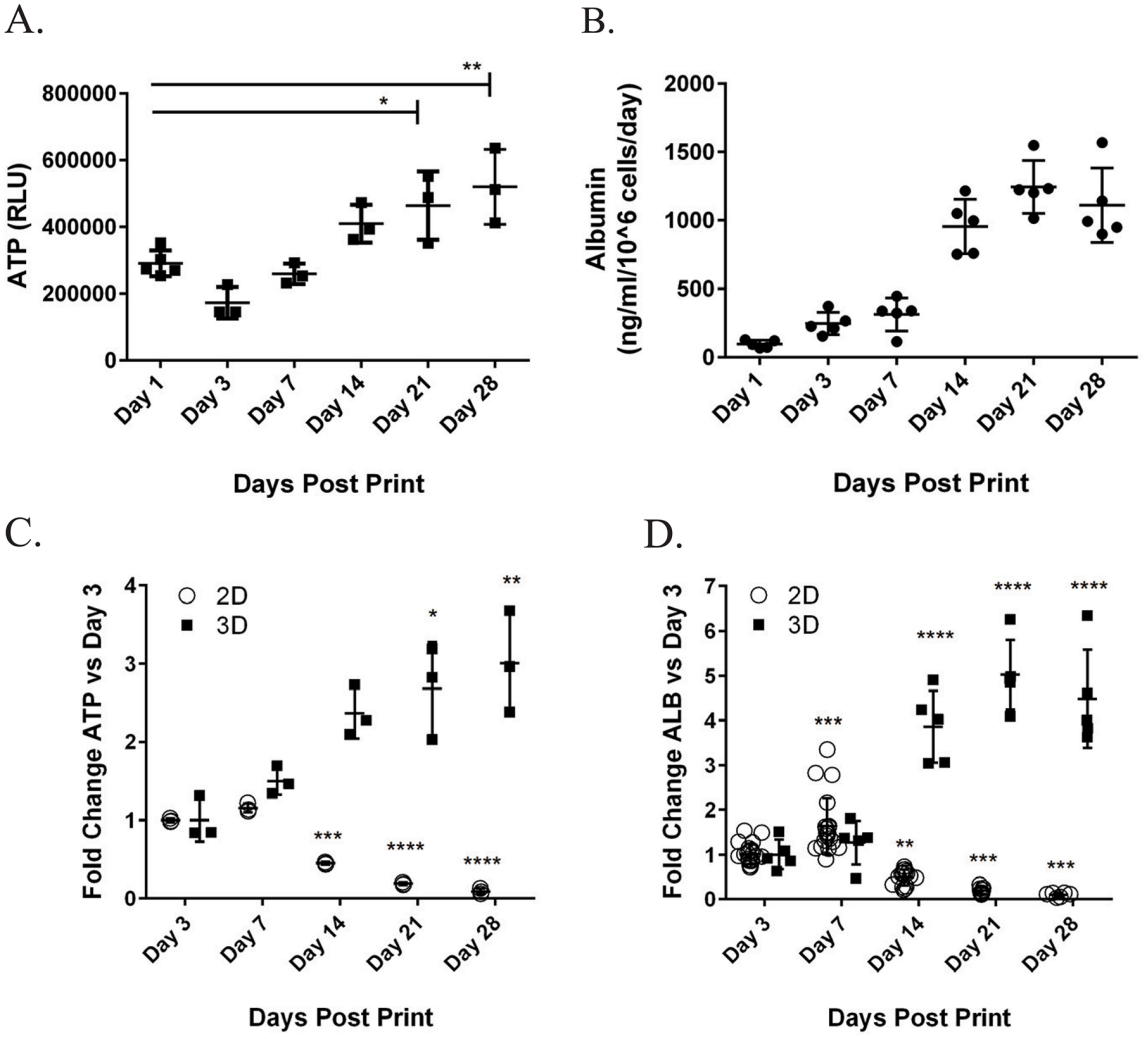
Measurement of tissue ATP and the secreted factor albumin from3D liver tissues over 28 days. A) Cell Titer Glo (Promega) was used to assess the levels of ATP in 3D liver tissues over time. Data shown is the average of 3 replicates +/- standard deviation. B) Albumin was measured in the supernatant of 3D liver tissues over 28 days in culture by ELISA. Data shown is the average of 5 replicates +/- standard deviation. C) Levels of ATP in 3D liver tissues (A) were compared to those from standard 2D hepatocyte culture over 28 days by normalizing to day 3 levels. Data shown is the average of 3 replicates +/- standard deviation. D) Levels of albumin (ALB) in 3D liver tissues (B) were compared to those from standard 2D hepatocyte culture over 28 days by normalizing to day 3 levels. Data shown is the average of at least 5 replicates +/- standard deviation. All statistics (One way ANOVA) and outliers (Grubbs’ test) were calculated using GraphPad Prism software; * p<0.05, ** p<0.01, ***p<0.001, **** p<0.0001.

To assess their general health, levels of ATP were measured in 3D liver tissues over 4 weeks. For the first two weeks in culture, ATP levels remained fairly consistent, with a statistically significant increase seen by Day 21 (Fig 5A). This increase could reflect enhanced metabolic capacity of the tissues, or continued proliferation of the non-parenchymal tissue components; ongoing proliferation of a subset of both cell types comprising the non-parenchymal compartment was verified by immunohistochemistry for proliferative cell nuclear antigen (PCNA), a marker of proliferating cells (S1 Fig). As an additional assessment of tissue viability, levels of secreted albumin were measured throughout the 4 week timecourse. Albumin, a marker of mature hepatocytes and general indicator of protein production capacity, was produced from the first day of fabrication and increased steadily until Day 14 (Fig 5B), reaching a plateau that was then maintained throughout the rest of the culture period. As suggested by the literature, this sustained pattern of albumin and ATP production is in sharp contrast to hepatocytes in standard 2D culture, which show precipitous declines in both ATP and albumin after two weeks and negligible levels of albumin by week 4 (Fig 5C).

**Fig 6:**
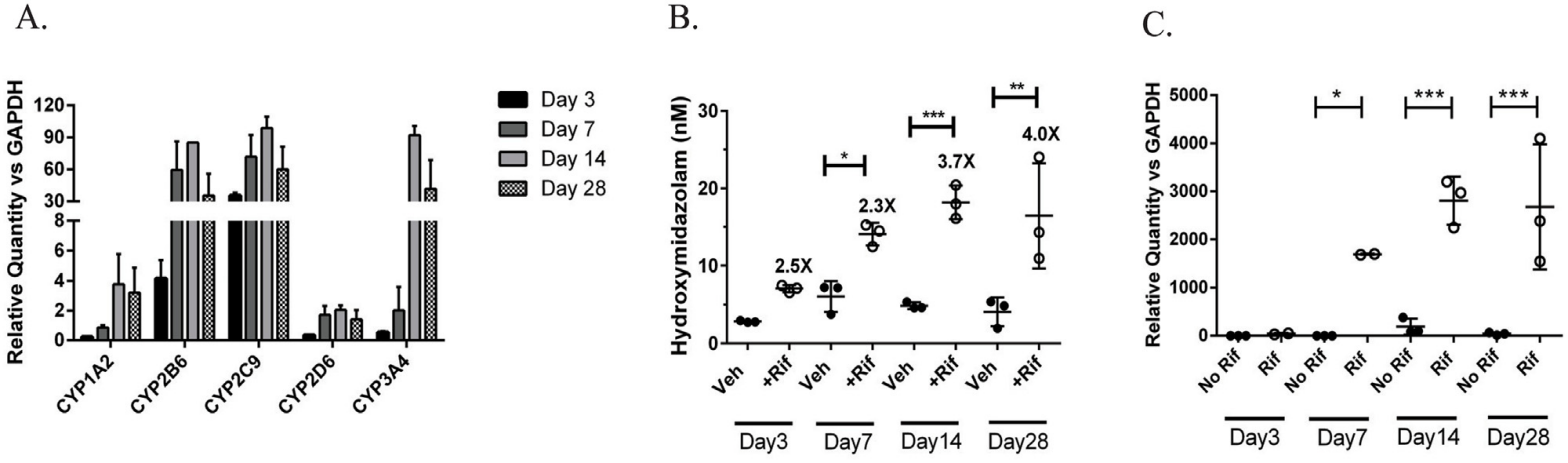
Expression of and function of CYP enzymes in 3D liver tissues. A) Quantitative RT-PCR was used to assess levels of CYPs 1A2, 2B6, 2C9, 2D6, and 3A4 in bioprinted liver tissues at the time points indicated. Data is expressed relative quantity (RQ) * 10000 after normalization to GAPDH, and is the average of 3 tissues +/- standard deviation. B) Basal and Rifampicin-induced CYP3A4 activity, measured by the formation of 4-hydroxymidazolam at the timepoints indicated, was determined using mass spectrometry. Data shown is the average of 3 replicates +/- standard deviation. C) Quantitative RT-PCR was used to assess levels of basal and Rifampicin-induced CYP3A4 in bioprinted liver tissues at the time points indicated. Data is expressed relative quantity (RQ) after normalization to GAPDH, and is the average of 3 tissues +/- standard deviation. All statistics (One way ANOVA) and outliers (Grubbs’ test) were calculated using GraphPad Prism software; * p<0.05, ** p<0.01, ***p<0.001, **** p<0.0001.

Cytochrome P450 enzymes (CYPs) are responsible for the first pass metabolism of xenobiotics, introducing small chemical changes that facilitate compound excretion. Temporal analysis of a CYP mRNA expression in the 3D liver tissues showed sustained basal expression of key CYP enzymes through 28 days in culture (Fig 6A). Interestingly, while levels of all enzymes increased during the first week of culture, expression of CYP3A4 and 1A2 did not reach a plateau until Day 14. This is in contrast to what has been seen with isolated primary hepatocytes, where they lose expression of this class of enzymes within two weeks in culture [11]. To verify enzymatic activity, the ability of tissues to metabolize the CYP3A4 substrate midazolam to 4-hydroxymidazolam was investigated. The 3D liver tissues were able to produce hydroxymidazolam throughout the 4 week culture period (Fig 6B). In addition, higher levels of metabolism were seen when CYP3A4 expression was further induced by exposure to Rifampicin, a well-known inducer of CYP enzyme expression [34]. The enhanced metabolic activity was accompanied by an expected increase in CYP3A4 mRNA (Fig 6C). Together, these results confirm the sustained viability and functionality of 3D liver tissues over time as well as their clear superiority over standard 2D culture.

### Trovafloxacin toxicity in 3D Liver Tissues

**Fig 7:**
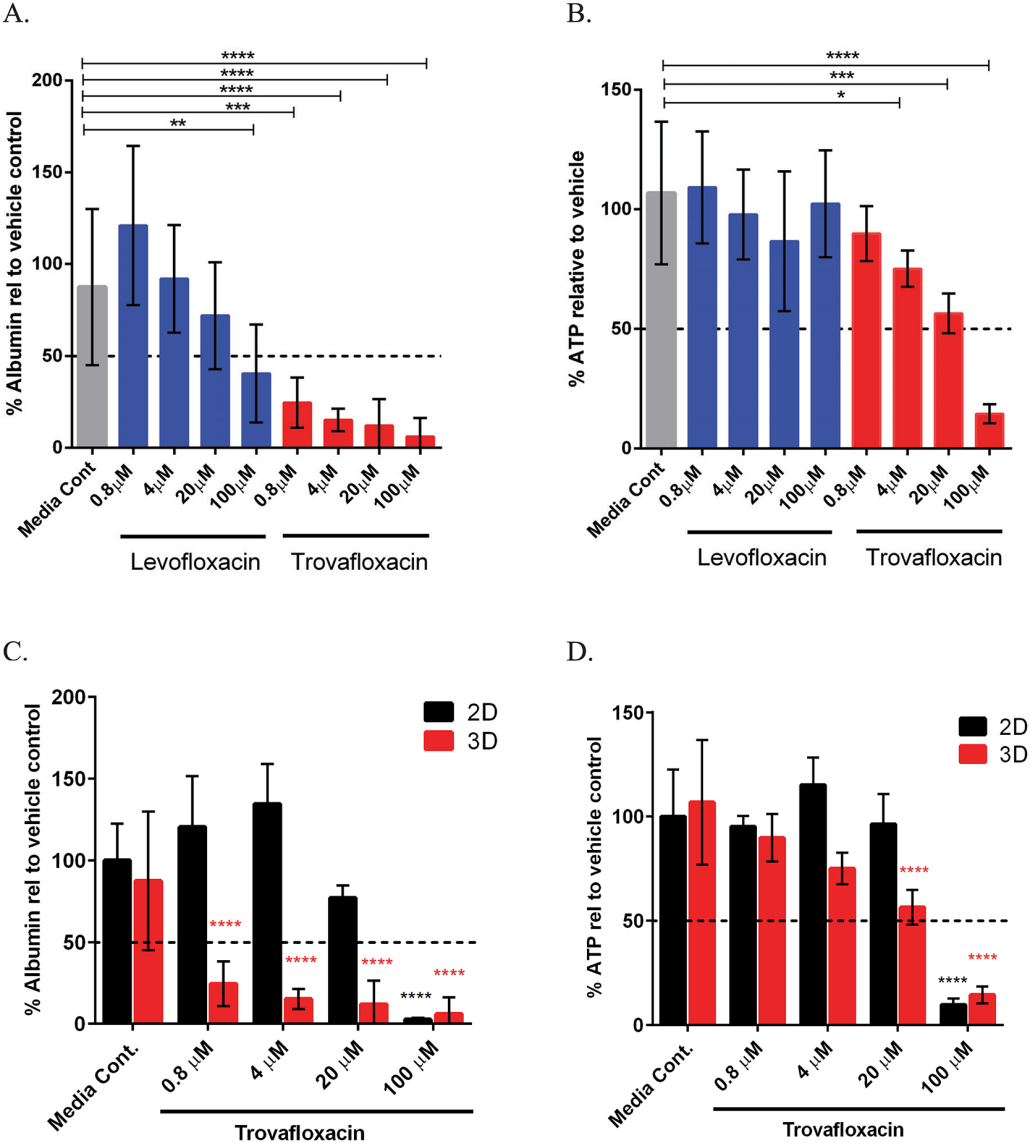
Biochemical Effects of Trovafloxacin and Levofloxacin in 3D bioprinted liver tissues. A) Measurement of secreted factor albumin in the supernatant of 3D liver tissues treated with Trovafloxacin or Levofloxacin daily for 7 days. Data is expressed as the percentage relative to the vehicle average and is the average of 10 replicates across two independent experiments +/-standard deviation. B) Measurement of ATP levels in 3D liver tissues treated with Trovafloxacin or Levofloxacin daily for 7 days. Data is expressed as the percentage relative to the vehicle average and is the average of 6 replicates across two independent experiments +/-standard deviation. C) Levels of albumin (ALB) in 3D liver tissues treated with Trovafloxacin (A) were compared to those from standard 2D hepatocyte cultures treated with Trovafloxacin daily for 7 days by normalizing to vehicle control. Data shown for 2D cultures is the average of 4 replicates +/- standard deviation. D) Levels of ATP in 3D liver tissues treated with Trovafloxacin (B) were compared to those from standard 2D hepatocyte cultures treated with Trovafloxacin daily for 7 days by normalizing to vehicle control. Data shown for 2D cultures is the average of 4 replicates +/- standard deviation. All statistics (One way ANOVA for 7A, B; Two way ANOVA for 7C, D) and outliers (Grubbs’ test) were calculated using GraphPad Prism software; * p<0.05, ** p<0.01, ***p<0.001, **** p<0.0001.

**Fig 8:**
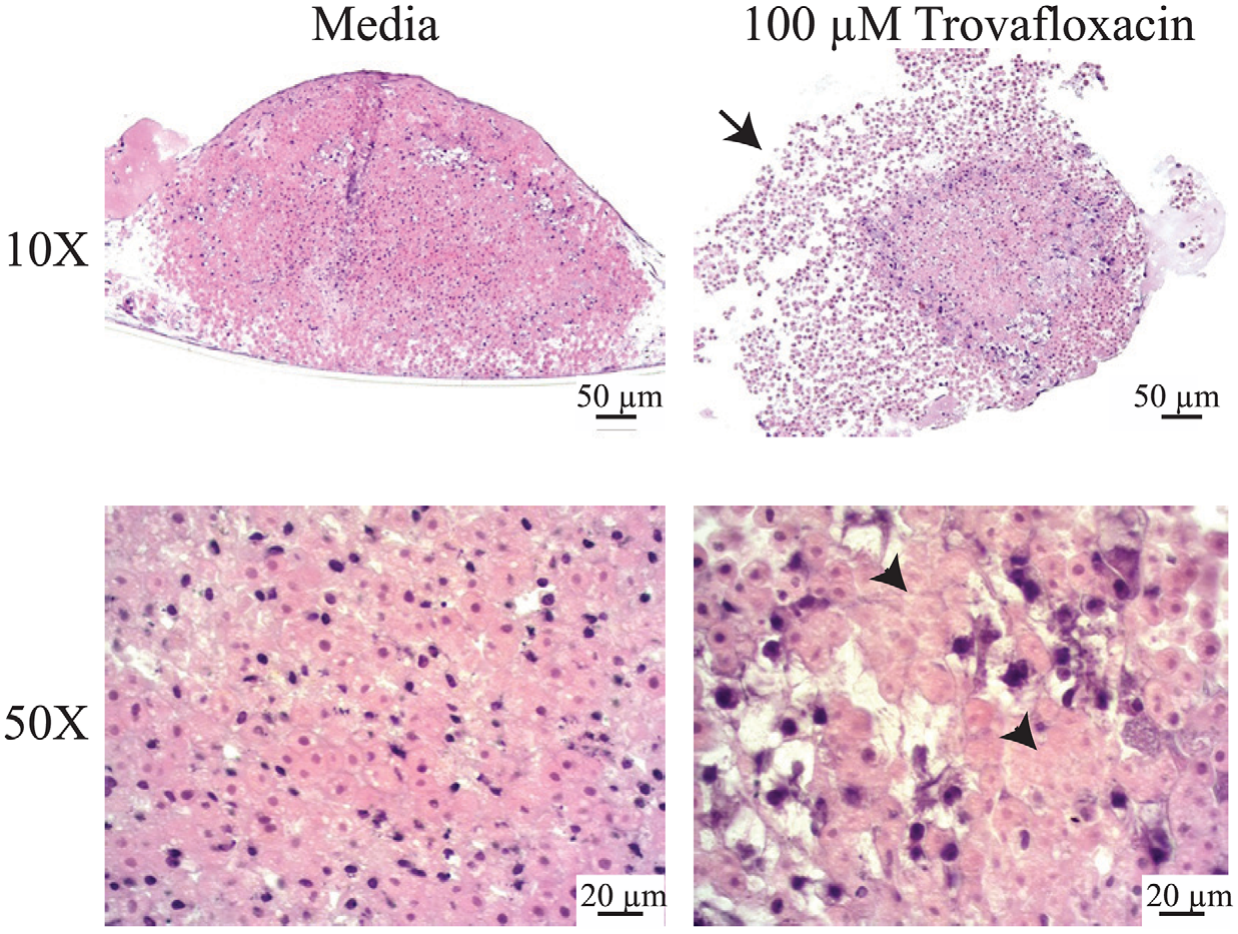
Histological Effects of Trovafloxacin in 3D bioprinted liver tissues. H&E staining of an untreated (Media) or 100 μM Trovafloxacin-treated 3D liver tissue, with cross-sections captured at either 10X or 50X magnification to visualize salient features. Histological analysis shows loss of cellular adhesion (arrow) and increased hepatocyte necrosis (arrowheads) in Trovafloxacin treated tissues.

Trovafloxacin is a third generation anti-infective, which received an adverse drug reaction (ADR) black box label and was subsequently withdrawn from the market one year following its approval due to liver failure and death in a small proportion of patients [35]. Interestingly, its hepatotoxic potential was not identified in pre-clinical models [36]. While the mechanism is not completely understood, Trovafloxacin toxicity is thought to have an inflammatory component [37]. This is largely based on data obtained in rodents after the drug was removed from the market, where induction of inflammation with lipopolysaccharide (LPS) enabled detection of toxicity following subsequent Trovafloxacin treatment [38]. The inflammatory component has been postulated to be mediated by Kupffer cells, the resident macrophages in the liver. However, multiple other non-parenchymal cell types can also produce inflammatory cytokines, including endothelial cells and hepatic stellates. Therefore, we investigated whether we could detect toxicity following treatement of 3D liver tissues withTrovafloxacin at doses previously shown not to induce toxicity in standard 2D culture [14], and compared these effects to the related non-toxic compound Levofloxacin. Following 7 days of dosing, Trovafloxacin induced significant decreases in both albumin (Fig 7A) and ATP (Fig 7B). The effects on albumin were more pronounced, with inhibition greater than 50% even at sub-micromolar doses. Levofloxacin led to a decrease in albumin only at the top dose tested (100 μM), and showed no effect on overall tissue ATP levels. In contrast, parallel 2D hepatocyte monocultures exposed to the same treatment conditions and timecourse only showed effects of Trovafloxacin toxicity at the highest dose tested (100 μM) regardless of the endpoint investigated (Fig 7C and 7D), further supporting the superiority of 3D liver tissues in predicting clinical hepatotoxicity. Considering that the levels of ATP and Albumin remain stable in the 2D hepatocyte cultures at Day 7 (Fig 5C and 5D), the lack of sensitivity of the 2D hepatocytes is likely not a consequence of decreased primary hepatocyte viability. Because the 3D liver tissues can be assessed histologically, we investigated the impacts of high dose Trovafloxacin versus vehicle on 3D liver tissues by histology. When compared to vehicle control, high dose Trovafloxacin decreased overall tissue cohesion and increased hepatocyte necrosis, leading to fragmentation (Fig 8). Notably, these effects were seen without any added inflammatory stimulus and in the absence of Kupffer cells.

## Discussion

Conventional cell culture models lack the complexity of native tissue and thus have a limited capacity for predicting tissue-level responses. The rapid loss of function in isolated human hepatocytes and species differences are major limitations in predictive toxicology which can lead to unexpected adverse outcomes in the clinic [10, 11]. We have developed a compartmentalized 3D human liver model, comprised of both parenchymal (hepatocyte) and non-parenchymal (HUVEC and HSC) cells. While they are not native to the liver, HUVEC cells provide a readily available, easily expandable source of endothelial cells; liver endothelial cells are a very specialized cell source that are difficult to obtain and expand. Fabrication of the liver tissues was enabled by additive manufacturing, which allowed the cellular inputs, spatial distribution, and geometry to be defined with high precision in the absence of exogenous scaffolds that can sometimes interfere with intercellular communication and alter the physiologic response to injury [22, 39]. Because the cells within the 3D structure are in close proximity to each other immediately after fabrication, they rapidly form tight junctions and deposit their own extracellular matrix, yielding solid microtissues that resemble native liver in relative cellular density. As highlighted by immunohistochemistry, tight junctions form between the hepatocytes, endothelial cells within the non-parenchymal zones migrate and organize, forming networks throughout the tissues, and the hepatic stellate cells localized to the tissue interior maintain a quiescent desmin positive, α-SMA negative state. While they are not native to the liver, HUVEC cells provide a readily available, easily expandable source of endothelial cells capable of forming microvasculature. Both glycogen storage and the accumulation of lipids were notable within the fields of hepatocytes in the 3D tissues, creating the potential to employ the tissues in chronic experiments that examine perturbations in these storage pathways over time. The overall tissue structure does not fully recapitulate the native liver lobule, however the presence and organization of multiple cell types within the compartmentalized structure of the 3D liver tissues likely play a significant role in preserving liver-specific functions, including stable levels of albumin and retention of basal and inducible expression and function of key CYP450 enzymes over 4 weeks. The general health of the tissues is further supported by the continued production of ATP over time. Comparisons to 2D co-cultures comprising all of the cell types included in the tissues are complicated by the rapid activation and hyperproliferation of HSC in 2D culture [40]. However, analysis of these same functions in “gold standard” hepatocyte 2D monocultures of the same donor hepatocytes demonstrates the clear enhanced longevity of these 3D liver tissues. While further characterization is needed, these observations support the use of 3D liver tissues to model the pathophysiologic effects of chronic dosing or conditions that develop over extended periods of time (i.e. steatosis, cholestasis, fibrosis, and infectious diseases such as hepatitis).

To determine the ability of the 3D liver tissues to model DILI, we investigated the effects of Trovafloxacin, a well-known inducer of hepatotoxicity which was withdrawn from the market due to induction of acute liver failure in a small proportion of patients [35, 41]. Trovafloxacin does not show strong toxicity signatures at clinically relevant doses in common in vitro systems, and toxicity is only seen in rodent models if an inflammatory stimulant like LPS is co-administered [37, 42]. These findings have led researchers to conclude that the liver effects of Trovafloxacin may be dependent on underlying inflammation mediated by Kupffer cells, the resident myeloid cell type in the liver. Interestingly, we saw marked toxicity in the 3D liver system without any exposure to LPS, and in the absence of Kupffer cells. These effects were seen at clinically relevant doses [14], were more evident in 3D tissues versus parallel 2D hepatocyte monocultures, and were detected both biochemically and histologically. While Kupffer cells can be isolated from human liver, they represent only a fraction of the liver mass and unlike stellate cells, they do not expand in culture. This makes it difficult to source enough cells from the same donor to perform multiple replicate studies. Different donor lot of Kupffer cells have been shown to provide a variable response to stimulation, which can lead to inconsistent results[43]. The ability to model the effects of compounds with an inflammatory component like Trovafloxacin without the need for Kupffer cells could provide a solution to these challenges. In addition, this data may suggest that other non-parenchymal cell types, such as endothelial cells or stellates, may be playing more of a role in Trovafloxacin toxicity than previously appreciated. By performing studies in 3D liver tissues with different cellular composition, mechanisms of hepatotoxins like Trovafloxacin could be more effectively prosecuted.

In summary, we have shown that this unique 3D model comprising multiple relevant liver cell types allows for the study of the tissue response to insult beyond simple cytotoxicity, enabling the measurement of cell type specific responses as well as in vitro histological assessment over extended time in culture. The benefits demonstrated in the 3D human liver tissues could be a result of the inclusion of the non-parenchymal cells, the 3D tissue context, and the compartmentalized architecture, or a combination of these features. Follow-up studies that attempt to dissect the contribution of each of these features will help us understand whether any one feature alone or in combination drives the enhanced capability of the model. In addition, a controlled system such as this where specific cell types can be added in or removed on demand may allow the specific contribution of each individual cell type to be defined for a given physiologic outcome. Future efforts will be focused on inclusion of some of these additional specialized liver cells (ex: Kupffer cells, sinusoidal endothelial cells, bile duct epithelium) in the 3D liver tissues, looking at their impact on both basic functionality and xenobiotic response.

## Supporting Information

S1 Fig Histological characterization of proliferation in 3D liver tissues. IHC staining for PCNA (green; nuclei of proliferating cells) and either Albumin, CD31 or desmin (red) suggests proliferation in a subset of the non-parenchymal cells. DAPI was utilized to stain the nuclei of the cells in all of the IHC staining samples (Blue). The dashed white lines show the division between the non-parenchymal compartment (NPC) and the hepatocyte-containing parenchymal compartment (PC). In the desmin / PCNA / DAPI stain, the image is focused on the NPC.
(TIF)
