# Supplementary figures and images for: Bioprinted 3D Primary Liver Tissues Allow Assessment of Organ-Level Response to Clinical Drug Induced Toxicity In Vitro
Source: PLoS One. 2016 Jul 7;11(7):e0158674. doi: 10.1371/journal.pone.0158674 (PMC4936711; doi:10.1371/journal.pone.0158674)

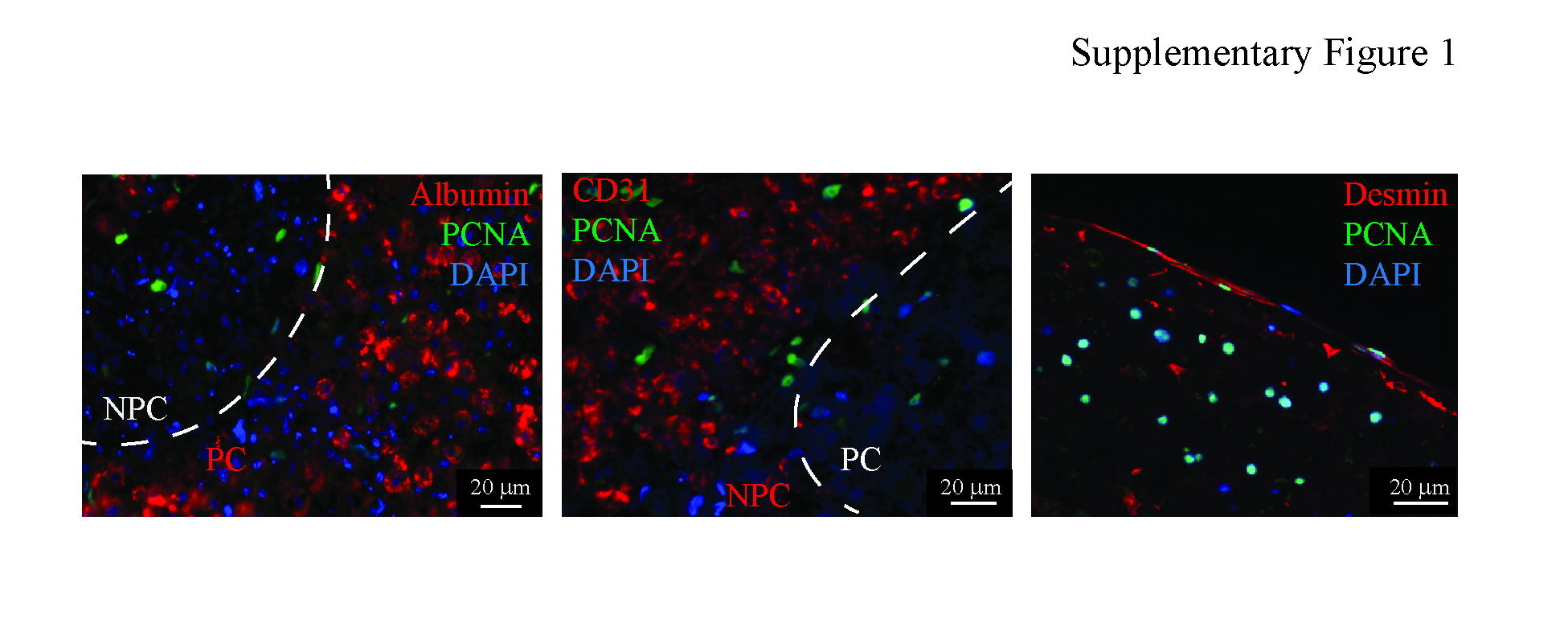

Supplement: S1 Fig — IHC staining for PCNA (green; nuclei of proliferating cells) and either Albumin, CD31 or desmin (red) suggests proliferation in a subset of the non-parenchymal cells. DAPI was utilized to stain the nuclei of the cells in all of the IHC staining samples (Blue). The dashed white lines show the division between the non-parenchymal compartment (NPC) and the hepatocyte-containing parenchymal compartment (PC). In the desmin / PCNA / DAPI stain, the image is focused on the NPC. (TIF) [file pone.0158674.s001.tif]
